# Mapping dynamics in neuronal activation and molecular connectivity with functional PET

**DOI:** 10.3389/fnins.2026.1920555

**Published:** 2026-09-28

**Authors:** Andreas Hahn, Murray B. Reed, Pia Falb, Matej Murgaš, Christian Milz, Sarah M. Tüchler, Sebastian Klug, Godber M. Godbersen, Rupert Lanzenberger

**Affiliations:** 1 Department of Psychiatry and Psychotherapy, Medical University of Vienna, Vienna, Austria; 2 Comprehensive Center for Clinical Neurosciences and Mental Health (C3NMH), Medical University of Vienna, Vienna, Austria

**Keywords:** dopamine synthesis, glucose metabolism, molecular connectivity, multimodal brain imaging, serotonin synthesis

## Abstract

The assessment of brain function using PET imaging has recently gained increasing interest due to several technical advancements, including bolus+infusion radiotracer administration protocols combined with hybrid PET/MR or high-sensitive PET/CT scanners. Functional PET (fPET) enables imaging of stimulation-induced changes in glucose metabolism and neurotransmitter action in a single scan as well as computation of molecular connectivity represented by temporal associations of PET signals between brain regions. In addition to methodological considerations, this work aims to highlight key advantages, limitations and future challenges of fPET and applications in multimodal imaging. Regional increases in glucose metabolism largely correspond to those observed with BOLD fMRI across a range of cognitive tasks, whereas divergence within the default mode network during internally directed attention seems to reflect active inhibitory processes. Extensions to radioligands beyond [^18^F]FDG demonstrated marked increases in dopamine and serotonin synthesis during reward processing, providing complementary insight into neurotransmitter action. Moreover, image reconstruction in the range of seconds facilitates extraction of moment-to-moment fluctuations in fPET signals for computation of molecular connectivity. The use of large axial field-of-view PET scanners for total-body imaging further enables inference on molecular interactions at the systems level. In sum, fPET imaging, particularly multimodal combinations, offers novel perspectives that cannot be obtained with either of these modalities alone. Future challenges include the detailed characterization of the relationships between neurovascular and neurometabolic coupling as well as the physiological underpinnings of rapid changes in fPET signals and the translation to clinical research settings.

## Introduction

The assessment of brain function represents a major cornerstone of neuroscience. In addition to electro- and magnetoencephalography (EEG, MEG), tomographic imaging techniques such as positron emission tomography (PET) and magnetic resonance imaging (MRI) have an essential role as they enable the non-invasive investigation of the living brain. These allow to derive specific changes in response to cognitive, pharmacological or neuromodulatory stimulation, a paradigm that has fundamentally advanced the understanding of the brain.

First imaging studies on brain function were conducted around 1980 with autoradiography (Sokoloff et al., 1977), scintillation detectors (Lassen et al., 1978) and PET as known today (Phelps et al., 1981) showing regionally specific changes upon stimulation (see Raichle, 2009 for a detailed historical review). These set the basis for the quantification of functional changes in cerebral blood flow (CBF) and the cerebral metabolic rate of glucose (CMRGlu) using [^15^O]H_2_O and [^18^F]FDG bolus applications, respectively. With the discovery of the blood-oxygen level dependent (BOLD) effect (Ogawa et al., 1990), functional MRI (fMRI) offered a particularly practicable alternative to the more complex PET experiments, without the need for tracer synthesis and application, no radiation exposure and thus overall lower costs, enabling broad access to functional brain imaging. As such, fMRI has remarkably improved the investigation of brain function. In addition, the use of resting-state fMRI (Biswal et al., 1995) paved the way for the computation of functional connectivity (Fox et al., 2005) and the investigation of brain network organization (Biswal et al., 2010; Buckner et al., 2008; Fox and Raichle, 2007). It is important to mention that the BOLD signal and PET-derived metabolic demands resemble different views on the same underlying phenomenon of neuronal activation, facilitated through neurovascular and neurometabolic coupling, respectively. While the BOLD effect is a composite of changes in blood flow, volume and oxygenation (Norris, 2006; Heeger and Ress, 2002; Attwell and Iadecola, 2002; Logothetis, 2008), glucose metabolism imaged with PET is more closely related to synaptic activity (Yu et al., 2018; Attwell and Laughlin, 2001; Harris et al., 2012; Mergenthaler et al., 2013). The development of hybrid PET/MR scanner systems has driven further progress in functional brain imaging by enabling the complementary assessment of brain function through simultaneous investigation of stimulus-evoked responses (Verger and Guedj, 2018). This work highlights the most recent development in functional PET with [^18^F]FDG imaging for identification of transient changes in metabolic demands. This includes its validation, application and multimodal combinations as well as extensions to neurotransmitter systems and high-temporal resolution imaging, the latter facilitating direct comparison of brain signals in the temporal domain and computation of molecular connectivity.

## Renaissance of functional pet with bolus + infusion protocols

As mentioned above, earlier studies employed a bolus application of [^18^F]FDG. In these paradigms, task performance at the beginning of the scan effectively leveraged the tracer’s irreversible uptake, such that stimulation-related effects remained detectable at later imaging timepoints. While completely valid and feasible, this approach requires repeated scans for rest and for each task condition, introducing potential variability in physiological states across scan session as well as increased radiation burden and experimental burden.

The concept of assessing task-related effects in a single scan emerged independently in two laboratories. Harvard University conducted the first proof-of-concept in 2014 with three individuals (Villien et al., 2014), introducing a constant infusion protocol combined with a general linear model (GLM) to separate task effects from baseline radiotracer uptake. In parallel, the Medical University of Vienna was able to later validate and refine the approach, providing full quantitative characterization in a larger sample and identification of multiple task conditions (Hahn et al., 2016). These studies demonstrated that visual and/or motor stimulation elicits regionally specific increases in glucose metabolism in corresponding primary sensory cortices, thereby establishing the feasibility of detecting within-scan functional modulation. Here, paradigms employed block designs of 5–10 min and GLM frameworks with regressors to characterize the different conditions (see below).

Considering its conceptual similarities to fMRI task design and analysis, the term functional PET (fPET) was established. It should be noted that several alternative approaches exist to assess functional changes with PET, such as multiple separate bolus scans (Phelps et al., 1981; Vlassenko et al., 2006) and multi-injection protocols (Nishizawa et al., 2001) for glucose metabolism as well as the competition model to assess changes in neurotransmitter release of the dopamine (Morris et al., 2024) and serotonin systems (Agnorelli et al., 2026). The present work, however, focuses on the developments in fPET using a bolus+infusion tracer administration for the assessment of human brain function. Still, we would like to emphasize that fPET has also been used in other species and organs, including neuroimaging in non-human primates (Villien et al., 2014) and rodents (Haas et al., 2024), as well as cardiac applications in pigs (Barton et al., 2018), thereby highlighting the versatility of the approach.

### Imaging protocol

As [^18^F]FDG is almost irreversibly trapped, any change in glucose metabolism results in a correspondingly changed slope of the time activity curve (TAC), which can easily be quantified with the Patlak plot (Hahn et al., 2016). To enable the detection of transient metabolic changes, early fPET studies employed a constant infusion protocol (without an initial bolus) that maintain a sustained availability of radiotracer throughout the scan, allowing for regional binding proportionally to the underlying metabolic demands (Villien et al., 2014; Hahn et al., 2016).

As a major advantage, the approach enables to quantify (at least) three different cognitive states in a single scan. The technique was then further enhanced with a bolus+infusion protocol, where 20% (Rischka et al., 2018) to 50% (Jamadar et al., 2022) of the total activity is used for the initial bolus and the rest for the infusion. This improves the signal to noise ratio particularly for early time frames and yields a stable (near-linear) uptake after the initial bolus has passed (Figure 1a). Thus, the bolus+infusion administration is the currently recommended approach.

**Figure 1:**
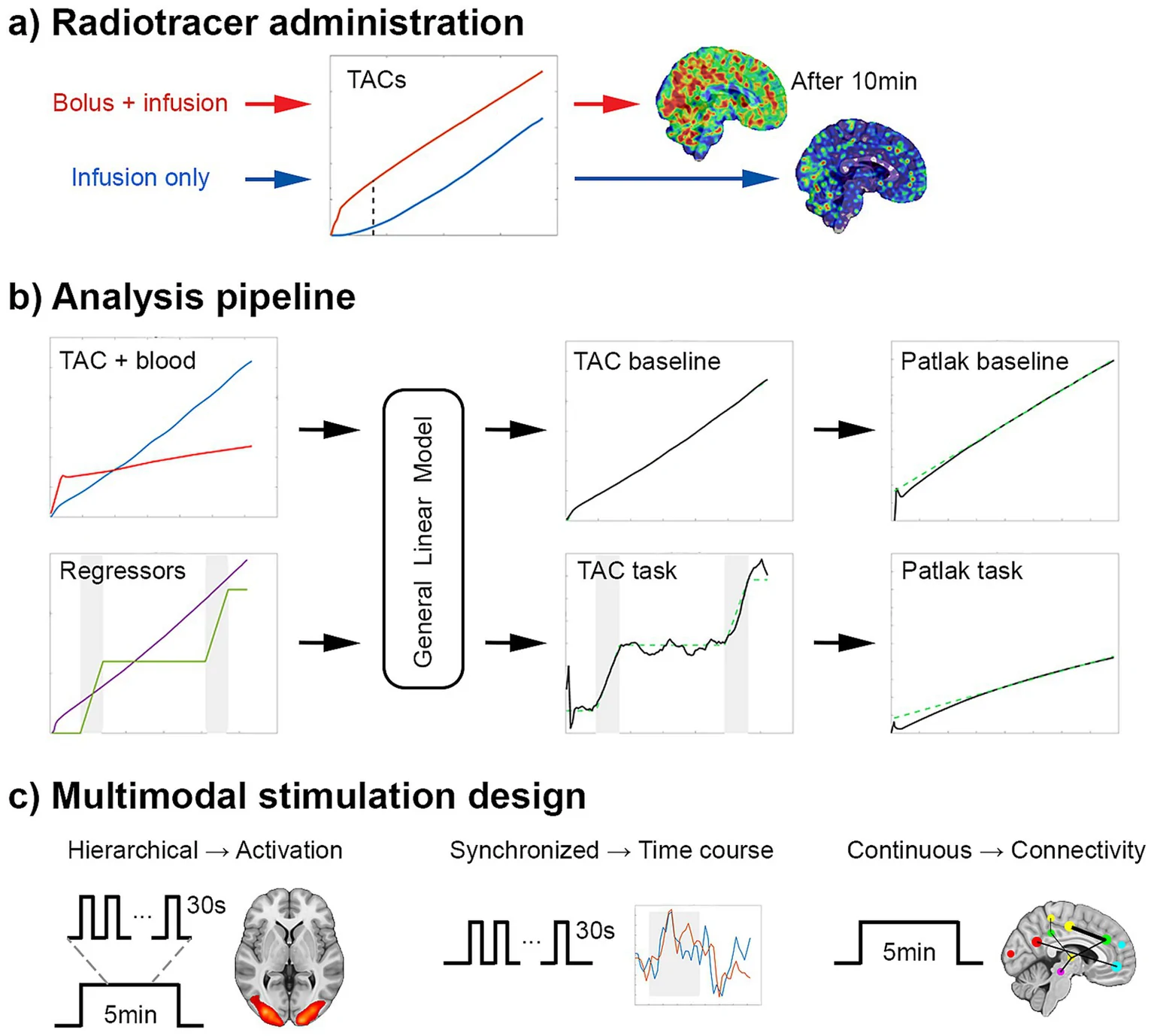
Design of fPET studies and analysis. **(a)** Radiotracer administration is carried out with an infusion only (blue) or combined bolus+infusion protocol (red). The latter yields substantially higher signal to noise ratio (SNR) of the resulting image frames, here shown for representative subjects after 10 min, as indicated by the dashed vertical line in the time activity curves (TAC). Note that the maximum scaling is 3 times higher for the bolus+infusion than for the infusion only images. Thus, the bolus+infusion protocol is the recommended and mostly used approach. **(b)** Data analysis can be realized with the general linear model (GLM). Input data are the TACs (blue) and regressors characterizing baseline and stimulation-induced radiotracer uptake. The GLM separates these two signal sources and subsequent application of the Patlak plot allows absolute quantification of changes in glucose metabolism. Gray areas indicate times of task performance. **(c)** For multimodal fPET/fMRI acquisition the stimulation design is optimized according to the research question. In a hierarchical design, short task blocks for BOLD fMRI imaging are embedded into longer task blocks for fPET. This design is often used for simultaneous assessment of neuronal activation. Fully synchronized investigation of the different signals employs conventional fMRI block designs, but requires reconstruction of fPET at high temporal resolutions in the range of seconds and sophisticated processing to compensate for the low SNR. This enables direct comparison between the different signals in the temporal domain. If task-specific functional connectivity is investigated, task presentation usually needs to be adapted to provide continuous stimulation periods without rapid on–off task switches for both modalities. Timings for fPET and fMRI indicate examples of common task durations. Data in **(a)** was taken from Jamadar et al. (2022), data in **(b)** was taken from Hahn et al. (2020), images in **(c)** was adapted from Hahn et al. (2023) and Klug et al. (2022), all under CC-BY license.

### Data analysis

The most widely used framework to assess changes in glucose metabolism with fPET is the GLM (Figure 1b). The design matrix includes a baseline term, one regressor for each task and nuisance variables. Unlike in fMRI, the baseline term must be actively defined within the GLM and aims to model resting state radiotracer uptake without contamination by task effects. It is often modeled as an average gray matter TAC (excluding voxels where activation is expected (Rischka et al., 2018)) or a third order polynomial (Hahn et al., 2016). While the former provides a better model fit, the latter is feasible if spatial extent and/or the shape of task effects are unknown (Rischka et al., 2018). However, a polynomial fit may introduce small but systematic bias in task estimates potentially leading to artifactual (de)-activations, although this seems to be a particular issue when combined with an infusion-only administration protocol without an initial bolus (Coursey et al., 2025).

Task regressors are usually modeled as a ramp function (i.e., an integrated boxcar function), with a slope of 1 kBq/min during stimulation and 0 otherwise. Despite its simplicity, this has been shown to yield robust activation across a wide range of cognitive paradigms (Villien et al., 2014; Hahn et al., 2016; Hahn et al., 2020; Jamadar et al., 2019; Stiernman et al., 2021; Putkinen et al., 2026). Nuisance variables can be optionally included to correct for head motion (e.g., as derived from realignment parameters) or other artifacts, thereby reducing false positive task estimates. Furthermore, improvement of data quality before modeling will also increase confidence in task estimates. Motion correction (Falb et al., 2026) and filtering or smoothing approaches (Reed et al., 2025c) are widely used, but more sophisticated techniques as used for fMRI (Glover et al., 2000) still need to be evaluated.

Quantification of CMRGlu is then mostly realized with the Patlak plot (Hahn et al., 2016) when an arterial or image-derived input function (Reed et al., 2023) is available. A non-invasive alternative is the calculation of percent signal change from baseline. This yields highly similar activation maps, thereby offering a valuable solution if arterial blood sampling is not feasible (Godbersen et al., 2024).

On the other hand, independent component analysis represents a data-driven alternative to assess stimulation-induced changes. This approach yields comparable activation patterns for both simple visual tasks (Li et al., 2020) as well as sophisticated optogenetic stimulation (Haas et al., 2024). It can be particularly relevant if task effects are unknown and has the advantage of removing noise components. However, the optimal preprocessing strategy and number of independent components for this approach has yet to be systematically assessed.

These various analysis solutions have recently been implemented into the dedicated fPET toolbox (Hahn et al., 2025).^1^ It is a freely available open-source package that allows to model stimulation effects with various different techniques and settings.

### Stimulation design

The described increase in increased SNR also allows shortening the task and thus frame duration from minutes for infusion-only protocols (Villien et al., 2014; Hahn et al., 2016; Jamadar et al., 2019) down to 12–30s for bolus+infusion applications (Rischka et al., 2018; Jamadar et al., 2021) and combined with sophisticated filtering (Reed et al., 2025c) even 3 s (Hahn et al., 2023). This in turn offers more flexibility in stimulation designs, which can then be optimized for multimodal combinations.

Long task blocks around 5 min provide particularly stable estimates of changes in glucose metabolism (Rischka et al., 2018) and good test–retest reliability when compared to fMRI using BOLD or arterial spin labeling (ASL) (Rischka et al., 2021). However, most paradigms were established with fMRI and are often designed in blocks of approximately 30s or even event-related, as longer task durations may overlap with physiological noise (Poldrack et al., 2011). One solution for simultaneous imaging is a hierarchical design, where short task blocks for fMRI are embedded into longer ones for fPET (Figure 1c). While this strategy is straightforward for block design paradigms such as visual stimulation (Jamadar et al., 2019), more complex event-based paradigms such as the monetary incentive delay task require more sophisticated designs (Hahn et al., 2021). In the latter example, events were manipulated across longer periods to obtain blocks biased to monetary gain (or loss) for fPET. While the hierarchical design is particularly suitable when fMRI is used as a proxy of neuronal activation, incorporating rest periods can underestimate the effect size of the fPET parameters, unless it is explicitly modeled. This type of modeling requires high temporal resolution.

Reconstructing images in the range of seconds in turn enables the implementation of a fully synchronized design (Hahn et al., 2023). In this setting, the task timings follow that of a conventional fMRI block design, with on–off phases of about 30s also for fPET (Figure 1c). To compensate for the lower SNR and short task blocks, various options such as a higher number of task blocks and filtering techniques (Reed et al., 2025c) are feasible to maintain stable task estimation. The approach allows the direct comparison of BOLD fMRI and [^18^F]FDG time courses and has demonstrated that changes in CMRGlu already occur within seconds after beginning and ending of task performance (Hahn et al., 2023). This is also in line with animal studies showing almost instant changes in extracellular ATP and glucose levels (Mann et al., 2021; Li and Freeman, 2015; Roche et al., 2011). These fast changes in the [^18^F]FDG signal seem to be mediated by rapid adaptation of blood–brain barrier (BBB) transport via the glucose transporter 1 protein and subsequent metabolism to meet acutely increased metabolic demands [for review see (Hahn et al., 2026)].

Alternatively, in case fMRI is used for the computation of task-related functional connectivity (FC), a continuous task design is favorable. Here, prolonged and continuous task performance is employed, i.e., without rapidly alternating task-rest periods in a longer stimulation block (Figure 1c). This is particularly useful for tasks such as visual stimulation, video games (Hahn et al., 2020) and when tasks can be easily adapted for prolonged stimulation. For FC, such an approach avoids rest-task state transitions thereby reducing false positive connectivity estimates (Ganger et al., 2015; Cole et al., 2019). This in turn also enables computation of molecular connectivity mapping (MCM) to infer directionality of connections (Riedl et al., 2016) (see below).

Thus, fPET is a particularly versatile technique, but design specifications need to be tailored to the research question at hand.

## Multimodal imaging with fPET

The facilitated assessment of stimulation-induced changes in CMRGlu renewed the interest for parallel imaging with other modalities, including BOLD and ASL fMRI (Figure 2). Direct comparison between these techniques has shown strong regional correspondence of activated brain regions across various different task settings, including visual and/or motor stimulation (Rischka et al., 2018; Jamadar et al., 2019), visuo-spatial motor coordination (Hahn et al., 2020), working memory (Stiernman et al., 2021; Hahn et al., 2023), music (Putkinen et al., 2026) as well as sleep (Chen et al., 2025). The observed changes in CMRGlu of about 20–25% from baseline are in the same range to those of CBF (Kessler, 2003; Fang et al., 2020; Mintun et al., 2001; Fox and Raichle, 1986), thereby matching earliest work that demonstrated a CMRGlu-CBF coupling mechanism during neuronal activation (Fox et al., 1988). The agreement between imaging modalities has also been extended to the temporal domain, showing particular correspondence of signals obtained with [^18^F]FDG and BOLD fMRI in the range of seconds (Hahn et al., 2023) to minutes (Chen et al., 2025). The latter has further been combined with EEG where changes in glucose metabolism and hemodynamics coincided with different non-REM sleep stages (Chen et al., 2025).

**Figure 2:**
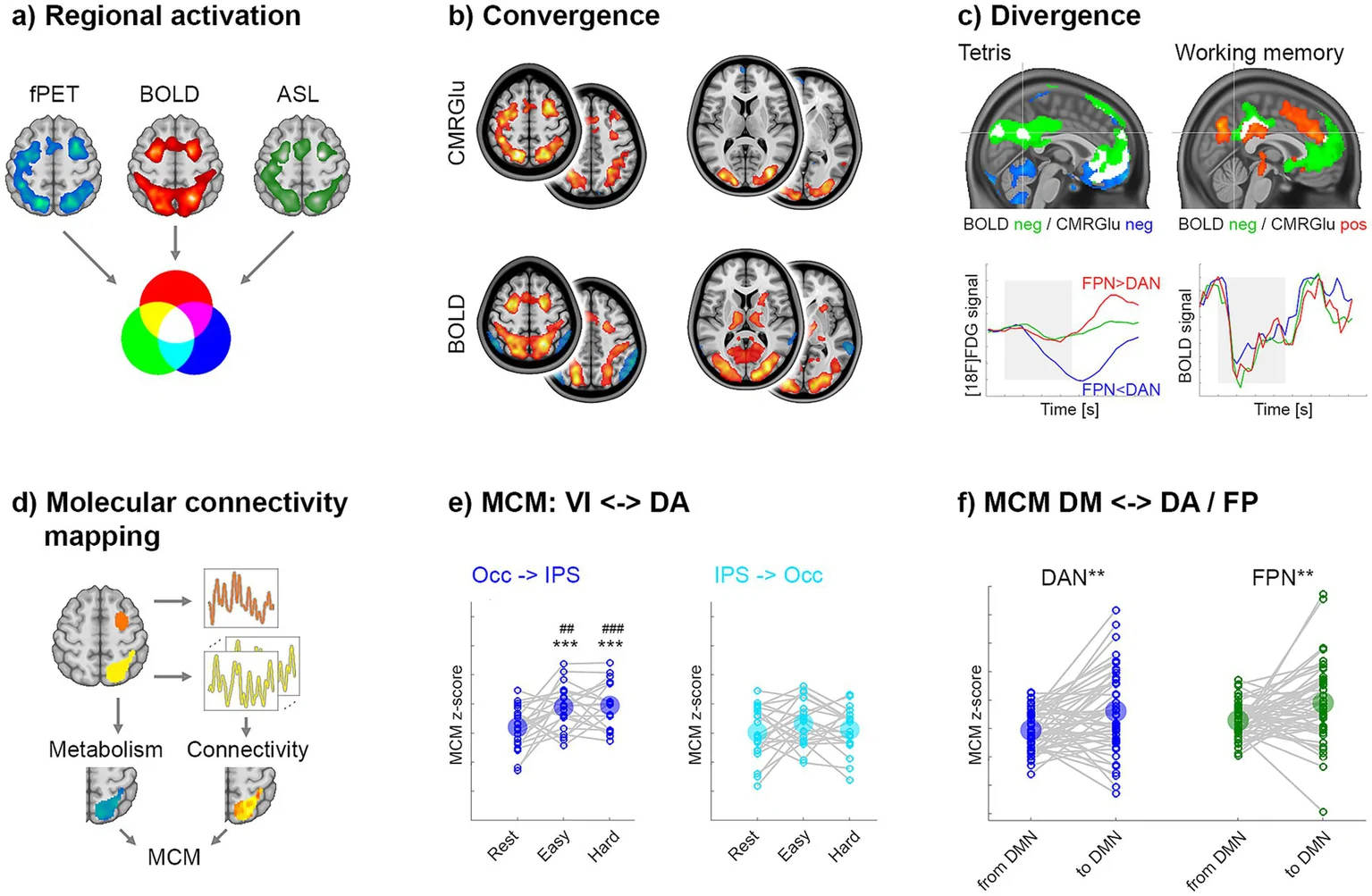
Exemplary multimodal combinations of [^18^F]FDG fPET and BOLD fMRI. **(a)** Schematic depiction to investigate the overlap of proxies of neuronal activation such as [^18^F]FDG fPET as well as BOLD and ASL fMRI. **(b)** Convergence between [^18^F]FDG fPET and BOLD fMRI has been observed for activations across a wide range of cognitive tasks. **(c)** In contrast, divergence has been repeatedly reported within the default mode network, particularly the posterior cingulate cortex (PCC). This applies to both the spatial (upper row) and temporal domains (lower row). **(d)** Schematic depiction of molecular connectivity mapping (MCM). Functional connectivity is computed from BOLD signals and the resulting pattern is spatially correlated with the corresponding CMRGlu pattern. Since metabolic demands emerge mostly postsynaptically, the target region and thus directional connectivity can be inferred. **(e)** Regions identified in **(b)** show increased MCM during task performance from the visual to the dorsal attention network. **(f)** The PCC identified in **(c)** receives task-specific input from the dorsal attention and frontoparietal networks. **(a,b,d,e)** were adapted from Hahn et al. (2020), **(c)** (spatial domain) and **(f)** were adapted from Godbersen et al. (2023) and **(c)** (temporal domain) was adapted from Hahn et al. (2023), all under CC-BY license.

The temporal domain of [^18^F]FDG signals has recently also been evaluated through its variation across time, calculated as the standard deviation of a denoised fPET time course (Deery et al., 2026). Denoising may include regression against signals from white matter and cerebrospinal fluid, which is analogous to component-based noise correction for molecular connectivity (Reed et al., 2026) (see below). The resulting glucodynamic variability has been shown to decrease with age, which was further linked to reduced cognitive performance (Deery et al., 2026), thereby offering a novel perspective to investigate time-varying metabolic demands.

Another promising multimodal combination is that of fPET with BOLD-derived functional connectivity, which allows to infer directionality of connections using molecular connectivity mapping (MCM) (Riedl et al., 2016). As the majority of energy demands occurs postsynaptically (Yu et al., 2018; Attwell and Laughlin, 2001; Harris et al., 2012; Mergenthaler et al., 2013) the target region of a corresponding connection can be identified. MCM computes the spatial association between voxel-wise patterns of glucose metabolism and functional connectivity. The underlying rationale is that if a connectivity pattern of a putative target region is causally related to the influence of another region, this yields a corresponding pattern of metabolism demands in said target region (Hahn et al., 2020; Riedl et al., 2016), due to the coupling of both signals to postsynaptic glutamate signaling (Attwell et al., 2010; Mishra et al., 2016). This approach successfully recapitulated hierarchical organization of major sensory networks at resting state (Riedl et al., 2016; Belenya et al., 2026). In addition, MCM showed that task performance leads to an increased association between functional connectivity and metabolism specifically for feedforward connections (Hahn et al., 2020), but decreases after prolonged learning of the task (Klug et al., 2022). The approach was further extended to model metabolic demands of a target region by a combination of functional inputs, with regional variation in explained variance correlating with non-oxidative energy metabolism and synaptic density (Klug et al., 2024).

Despite the largely reported concordance in task-induced activations between [^18^F]FDG fPET and BOLD fMRI, it has been shown that the two signals can also dissociate in meaningful ways, particularly in posteromedial regions of the default mode network. Here, well-known decreases in the BOLD signal were accompanied by unchanged or even increased glucose metabolism during working memory (Stiernman et al., 2021) and music (Putkinen et al., 2026), but not Tetris (Godbersen et al., 2023). This effect was further detailed to be related to the specific tasks at hand (internal vs. external attention) and the correspondingly activated networks, with DMN metabolism being inversely influenced by metabolic demands of the frontoparietal and dorsal attention networks (Godbersen et al., 2023). This was also replicated in the temporal domain with specific changes in the [^18^F]FDG signal for individuals showing high or low metabolic demands in dorsal attention and frontoparietal networks, respectively (Hahn et al., 2023). One possible interpretation is that dissociations between these signals are related to active inhibition by GABAergic neurons, which is energy demanding but still decreases the BOLD signal (Harris et al., 2012; Hu et al., 2013; Gu et al., 2019; Koush et al., 2021). On the other hand, concordant decreases in both signals seem to be mediated by decreases in glutamate signaling (Attwell et al., 2010; Lundgaard et al., 2015; Zimmer et al., 2017). It is worth to note that the posterior cingulate cortex is a cytoarchitecturally and functionally heterogeneous region (Vogt et al., 2006; Leech and Sharp, 2014) with different subparts being involved in both internally and externally directed tasks, respectively (Leech and Sharp, 2014; Leech et al., 2011). Thus, the combination of fPET and fMRI enabled to further disentangle neurovascular and neurometabolic underpinnings of posterior DMN function. Another dissociation has been described for older adults also for regions involved in cognitive control (Stiernman et al., 2023). Here, stronger increases of BOLD contrasts in elderly compared to young adults were not correspondingly matched by glucose metabolism, indicating that such overactivations in older subjects may not be of neuronal origin.

To sum up, multimodal imaging offers a complementary viewpoint, with fPET allowing novel interpretations that would not be possible to derive with each of these techniques alone.

## fPET for neurotransmitter action

In addition to the assessment of glucose metabolism, fPET has been successfully extended to neurotransmitter systems. The technique aims to image activity-dependent regulation of neurotransmitter synthesis pathways, thus referring to the term synthesis model. This can be seen complementary to the established competition model, which infers neurotransmitter release from changes in radioligand binding at postsynaptic receptors.

The mentioned neurotransmitters cannot pass the BBB, but rather are synthetized from precursor molecules by specific enzymes. The main pathway of dopamine synthesis is the conversion of tyrosine to dihydroxyphenylalanine (DOPA) via the enzyme tyrosine hydroxylase, and then to dopamine by aromatic amino acid decarboxylase (AADC). On the other hand, the enzyme tryptophan hydroxylase converts tryptophan to 5-hydroxytryptophan (5-HTP), which is further converted to serotonin by AADC. The approach leverages the use of radiotracers such as 6-[^18^F]FDOPA and [^11^C]AMT as surrogates of the precursor molecules DOPA and tryptophan, respectively. Similar to the precursors, also the radiotracers are incorporated into the neurotransmitter synthesis chain as they represent substrates for the corresponding enzymes, representing established approximations for dopamine (DeJesus, 2003; Doudet et al., 1998) and serotonin synthesis rates (Chugani and Muzik, 2000; Visser et al., 2011).

The crucial point is that stimulation-induced neurotransmitter release will increase the activity of the mentioned enzymes in order to re-fill the synaptic vesicles with *de novo* synthetized neurotransmitter. Tyrosine and tryptophan hydroxylase activities as well as AADC increase during neuronal stimulation (Morgenroth 3rd et al., 1974; Hamon et al., 1979; Barrio et al., 1997). Furthermore, dopamine synthesis is increased by amphetamine-induced dopamine release (Kehr et al., 1977; Hartvig et al., 1997), which also applies to serotonin synthesis and fenfluramine (Yamane et al., 1999). The incorporation of the mentioned radioligands into these regulatory chains will also increase their uptake into neuronal cells. Therefore, the change in the acquired fPET signal is presumably related to the amount of released neurotransmitter and hence to the applied stimulus (Figure 3). As neurotransmitter synthesis mostly occurs at nerve terminals, this allows for a topological identification of stimulation effects. Using the same principles of radiotracer administration, task design and analysis as described above, the approach has been successfully employed with fPET during reward processing, with changes of about 100–150% from baseline for dopamine (Hahn et al., 2021) and 20–40% for serotonin synthesis rates (Hahn et al., 2024).

**Figure 3:**
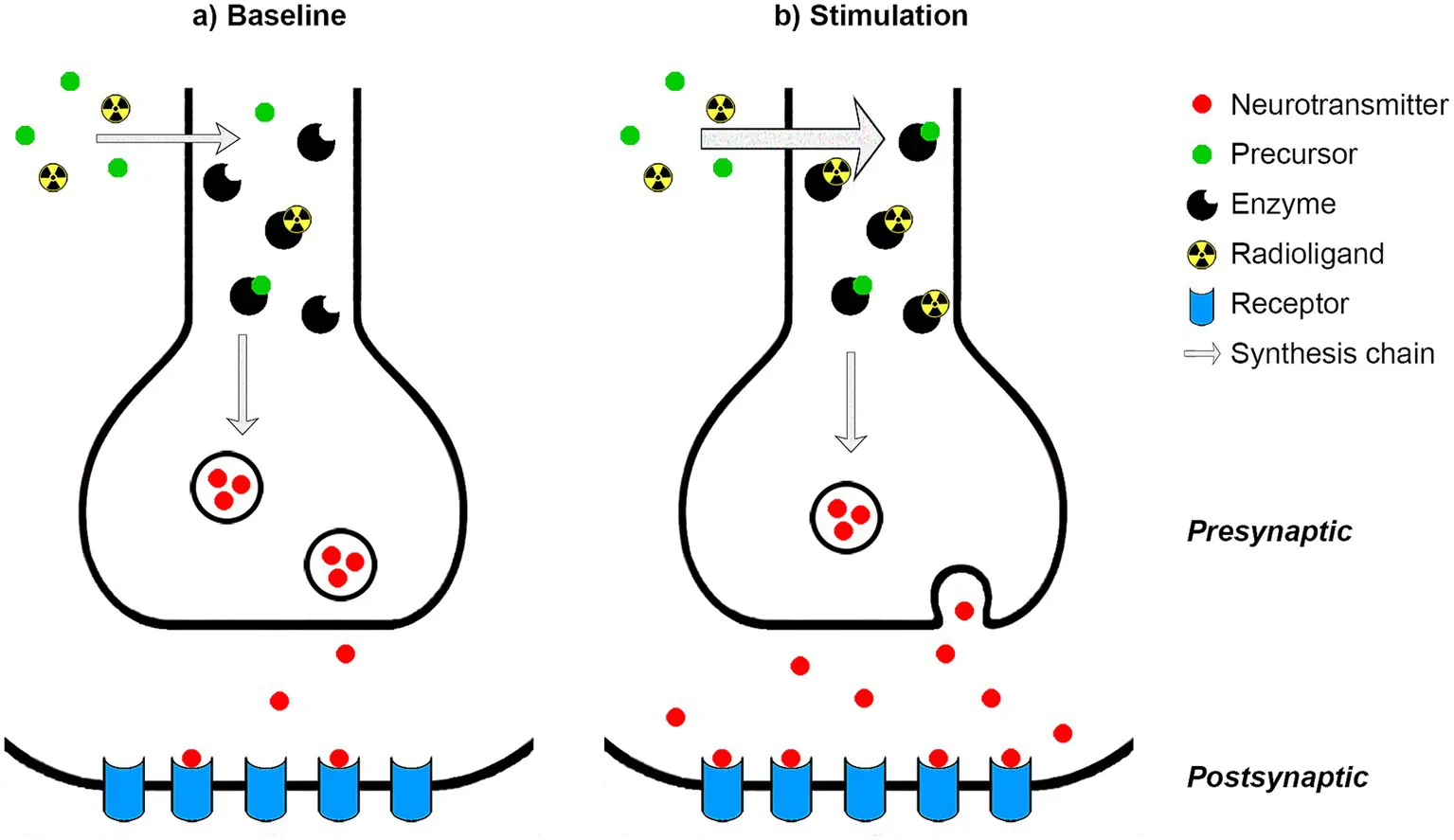
Schematic description of the synthesis model and its underlying biological basis. **(a)** The neurotransmitters dopamine and serotonin are synthesized from precursor molecules (tyrosine - > DOPA - > dopamine/tryptophan - > 5-hydroxytryptophan - > serotonin) by corresponding enzymes (tyrosine hydroxylase + AADC/tryptophan hydroxylase + AADC). The radioligands 6-[^18^F]FDOPA and [^11^C]AMT, respectively, are incorporated into this chain and provide established proxies for neurotransmitter synthesis rates. **(b)** Neuronal activation leads to neurotransmitter release, which in turn increases neurotransmitter synthesis in order to re-fill the synaptic vesicles. This leads to a correspondingly increased radiotracer uptake as indicated by arrow thickness. For simplicity only one enzymatic conversion is shown, but this may include two steps. Adapted from Hahn et al. (2021) and Hahn et al. (2024) under CC-BY license.

As a limitation, the exact mechanism and numerical relationship between neurotransmitter synthesis and release still needs to be determined in future work. We further acknowledge that the proposed approach is still an indirect measure of the endogenous neurotransmitter by drawing inference from stimulation-induced changes in neurotransmitter synthesis rates. This mechanism is supported by the abovementioned literature, demonstrating that neurotransmitter synthesis is subject to specific regulatory mechanisms, including neuronal activation and the relation to neurotransmitter release. The approach may thus open future possibilities for specific investigation in psychiatric and neurological disorders.

As mentioned above, the technique should be seen in addition to (not instead of) the competition model, providing an alternative approach to investigate neurotransmitter action. As the competition model leverages on the sensitivity of radioligands to endogenous neurotransmitter levels at postsynaptic receptors (Morris et al., 2024; Agnorelli et al., 2026), the combination of the two approaches would provide even more detailed information regarding two distinct but complementary aspects of neurotransmitter signaling. These are the dynamic regulation of enzymes responsible for neurotransmitter synthesis and the synaptic availability of endogenous neurotransmitter, respectively, which are both induced by neurotransmitter release.

## Molecular connectivity and covariance

The investigation of interactions between brain regions with PET emerged shortly after the introduction of task-based studies (Horwitz et al., 1984; Horwitz et al., 1995; McIntosh et al., 1994). Early work characterized brain network organization by computing interregional covariance matrices from static PET data, acquired across groups of subjects. The approach entails minimal experimental complexity, as regional metrics such as standard uptake values are correlated across individuals to identify patterns of coordinated variation between brain regions. Owing to its methodological simplicity, this approach requires only a single static PET acquisition and can therefore be readily applied to routinely acquired clinical data. Consequently, molecular covariance analysis remains widely used (Hahn et al., 2010; Pereira et al., 2018; Veronese et al., 2019).

A considerable limitation of metabolic covariance approaches is that network estimates are derived from intersubject variability and are therefore inherently defined at the group level. As a result, connectivity measures cannot be directly estimated for individual subjects without reference to a larger population. To address this limitation, several methods have been proposed to derive subject-specific information from group-based covariance networks, including jackknife perturbation techniques that quantify the contribution of individual participants to an underlying reference network. Nevertheless, the resulting metrics remain dependent on the characteristics of the reference cohort and should not be interpreted as direct measures of within-subject connectivity (Reed et al., 2025a).

The advent of bolus+infusion administration protocols has enabled image reconstruction at high temporal resolution, down to the order of seconds. This temporal information allows the characterization of dynamic fluctuations in PET signals between brain regions within individual subjects (Figure 4). Conceptually, this technique is analogous to BOLD-derived FC and, thus, the term “molecular connectivity” has been proposed to describe connectivity measures derived from temporally resolved PET signals estimated at the individual level (Reed et al., 2025a). Importantly, the slow cumulative baseline uptake must first be separated from more rapid signal fluctuations that are presumed to reflect dynamics in tracer kinetics. Several preprocessing strategies have been proposed for this purpose, including spatio-temporal filtering (Jamadar et al., 2020), frequency domain filtering (Reed et al., 2026), polynomial detrending (Reed et al., 2023) and regression of signals derived from white matter and CSF using component-based noise correction (CompCor) adapted from fMRI (Reed et al., 2026). Filtering and CompCor are also relevant for denoising of fPET signals. While this step currently includes motion parameters only as additional measure, further work is required to assess if the broad repertoire available for fMRI, such as ICA-based approaches (Li et al., 2020) as well as correction for cardiac and respiratory signals (Glover et al., 2000), also improves fPET analysis. Following preprocessing, molecular connectivity can be estimated using correlation-based or related network analytical methods.

**Figure 4:**
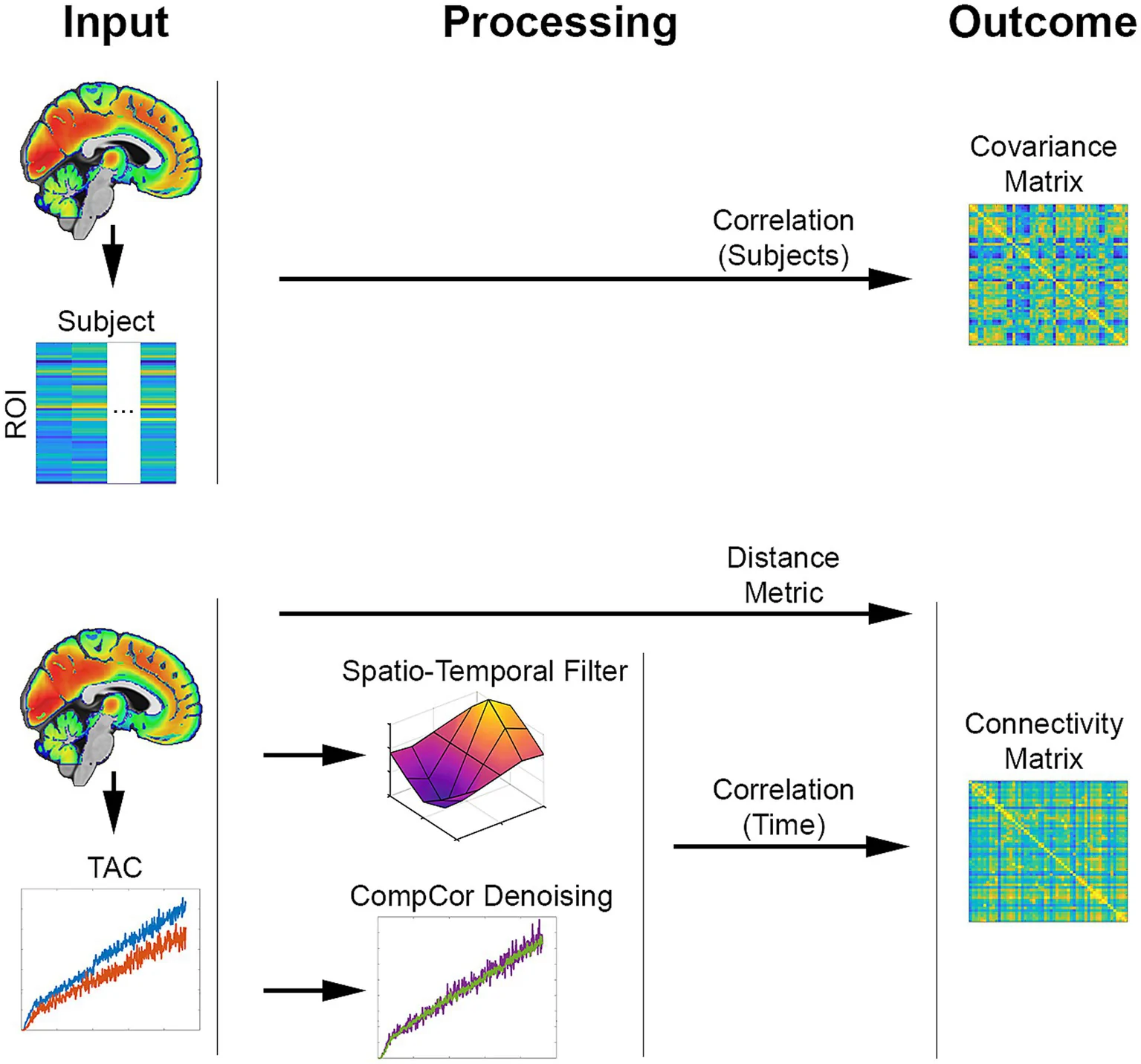
Molecular covariance and connectivity. If static PET data is available, this can be used to obtain a molecular covariance matrix (upper panel). Values such as standardized uptake values are extracted for each brain region and subject. Calculation of the pairwise regional correlations across subjects yields a covariance matrix. Due to its simplicity and widespread applicability the approach is particularly feasible. Interactions between brain regions are inferred at the group-level and potential individual contributions need to be done with reference to this group. Dynamic PET data allows computation of subject-level molecular connectivity (lower panel). Here time activity curves (TACs) of brain regions undergo, e.g., spatio-temporal filtering (Jamadar et al., 2020) or component-based noise correction (Reed et al., 2026). This removes the continuous radiotracer uptake and allows correlation of moment-to-moment fluctuations if data is reconstructed at sufficiently high temporal resolution (usually 1–16 s). Another approach that does not require signal preprocessing is to compute the Euclidean distance metric between TACs (Volpi et al., 2023) as a proxy of molecular connectivity. The spatio-temporal filter was adapted from Jamadar et al. (2020) under CC-BY license.

These approaches offer a new framework for investigation of brain network organization from a molecular perspective. Initial studies have reported alterations in molecular connectivity associated with healthy aging (Deery et al., 2024), while other work has extended the approach to the investigation of brain–body interactions using total-body PET imaging (Reed et al., 2023). As these are based on metabolic demands, they yield distinct and complementary information compared to functional connectivity (Reed et al., 2026; Deery et al., 2025). Furthermore, with the unique total-body systems, the investigation of high-temporal brain-organ connectivity can only be investigated using fPET.

Finally, an approach in-between the two former has been developed (Volpi et al., 2023). The method exploits dynamic PET data while avoiding explicit estimation of temporal correlations. One example computes the Euclidean distance between regional TACs at the individual level, quantifying the similarity of tracer kinetics across brain regions. Therefore, it can be applied to a wide range of administration protocols and temporal resolutions. Initial applications showed widespread alterations in metabolic network structure during task performance (Vallini et al., 2026) and glioma patients (Vallini et al., 2025).

Although comparisons between the different approaches have started (Reed et al., 2025b), the relative advantages, limitations, and optimal applications of these methods remain incompletely understood. Further methodological work is required to determine how the resulting network measures relate to one another and to the underlying biological processes they are intended to characterize.

## Limitations, outlook, and conclusion

Functional PET imaging offers investigation of human brain function from a molecular perspective. The recent development of bolus+infusion protocols enables assessment of stimulation-induced changes in glucose metabolism and neurotransmitter synthesis within a single scan as well as computation of molecular connectivity. Multimodal imaging with BOLD fMRI and/or EEG further allows to disentangle neurovascular and neurometabolic coupling mechanisms that shape neuronal activation and network organization. This represents a particular gain where the combination of the individual techniques yields more than the sum of its parts and novel insights which either modality alone could not determine.

fMRI offers excellent assessment of brain function both at high spatial (submillimeter) and temporal resolution (100–500 ms) by use of sophisticated sequences. These enable imaging of subcortical nuclei, cortical layers (Beckett et al., 2020; Townsend et al., 2025) and brain fluid dynamics (Kiviniemi et al., 2016), which are also available for conventional 3 T scanners. On the other hand, also PET experiences major advancements in scanner technology, such as the introduction of systems with a large axial field of view (Zhang et al., 2020; Prenosil et al., 2022) and brain-dedicated systems like the NeuroExplorer (Volpi et al., 2026) and UHR scanners (Doyon et al., 2025). These provide substantially improved spatial resolution (1.25–2.6 mm) as compared to conventional PET (Van Laere and Van Weehaeghe, 2026) and enable assessment of subcortical structures at unprecedented detail (Volpi et al., 2026; Doyon et al., 2025). Moreover, the up to 10-fold increased sensitivity of large axial field of view systems and the subsequently enhanced SNR allow for identification of novel insights in the temporal domain, such as cardiac pulsation (Zhang et al., 2020). This opens the application to high-temporal resolution fPET. Initial work has demonstrated the ability to reconstruct and underlined the overall validity of fPET data in the range of seconds, enabling further investigations of the relationships between [^18^F]FDG and BOLD signals (Hahn et al., 2023) as well as metabolic connectivity (Reed et al., 2026; Jamadar et al., 2020). However, the gain in temporal resolution also introduces new challenges and open questions that need to be addressed accordingly as the field continues to grow. For instance, the neurophysiological underpinnings of rapid changes and moment-to-moment fluctuations in the [^18^F]FDG signal are still not fully understood. Recent data and simulations indicate that blood–brain barrier transport of glucose plays an important role for stimulation-induced activation and subsequent phosphorylation (Hahn et al., 2026) due to the limited storage capacity of the brain, e.g., via glycogen. While task-specific changes in fPET signals seem to be largely independent of blood flow, its contribution to molecular connectivity still needs to be clarified (Hahn et al., 2026). Another limitation of current fPET analyses is the lack of knowledge of a metabolic response function, i.e., a molecular counterpart of the hemodynamic response function. That is, signal responses are usually modeled with a simple ramp function. Although this has provided robust task estimates, a more nuanced characterization would improve individual model fits and also offer a detailed investigation of the neurophysiological players of brain metabolism. At the group level statistical analysis for fPET is currently carried out with conventional software packages such as SPM and FSL. Although these adequately correct for the multiple comparison problem of voxel-wise analyses, accounting for PET-specific noise properties may further improve accuracy. This is however not unique to fPET, but applies to PET analysis in general. An aspect related to group analysis is the sample size of (f)PET studies, which is often smaller than in fMRI, due to cost, experimental complexity and regulations regarding radiation protection. Here, initiatives and databases such as the Alzheimer’s Disease Initiative, the EBRAINS infrastructure of the Human Brain Project and several PET-specific data sharing projects will help to push the sample size for such analyses, similar to what has been achieved with MRI-based platforms like the UK Biobank and the Human Connectome Project. An increased sample size would also facilitate deep-phenotyping studies, where a large number of subjects are required to enable application of machine learning techniques. As the above initiatives demonstrate that these efforts are feasible, adoption to fPET may further increase our knowledge of disease mechanisms. Compared to fMRI, fPET is however limited by the exposure of participants to radioactive isotopes. As regulations differ across countries, one to three scans with [^18^F]FDG are usually possible. Here, the use of high-sensitivity scanner systems (Prenosil et al., 2022; Al Sawafi et al., 2025) or deep learning approaches (Akita et al., 2025) enable substantial reductions of the required dosage, thereby allowing for an increased number of acquisitions.

Another advantage of the mentioned total-body scanner systems is the simultaneous assessment of multiple organs. In this context, functional PET offers the possibility to investigate system-level responses to stimulation such as heart-brain and gut-brain axes. fPET imaging already showed coupling of myocardial metabolism and ventricular function during hypoxic stress (Barton et al., 2018) and connectivity demonstrated distinct metabolic (Reed et al., 2023; Geist et al., 2025) and immunological (Maccioni et al., 2026) relationships between peripheral organs and the brain. On the other hand, smaller imaging systems developed for brain imaging such as NeuroLF (Mikhaylova et al., 2025) and wearable PET (Liu et al., 2026) may show particular clinical relevance due to decreased costs. Combination of these with fPET imaging enables novel investigation in more naturalistic stimulation settings. However, clinical applications of the described approaches are generally still scarce and translation of the technique represents a current challenge of the field. Several clinically relevant fPET studies assessed metabolic dynamics in in aging (Stiernman et al., 2023), particularly regarding the investigation of molecular connectivity (Deery et al., 2024; Deery et al., 2025). As connectivity and covariance approaches do not require stimulation equipment, these have also been applied in glioma patients (Vallini et al., 2025; Vallini et al., 2025), Parkinson’s disease (Heinecke et al., 2025) and to assess metabolic whole-body interactions across cancer, obesity and inflammation (Geist et al., 2025). Furthermore, fPET studies to evaluate consciousness in coma patients with auditory and emotional stimulation are currently performed (Godbersen et al., 2024; Hahn et al., 2025; Vallini et al., 2025; Geist et al., 2025; Paunet et al., 2023). A specific area of interest includes the biological interpretation of altered fMRI signals in psychiatric and neurological disorders through simultaneous assessment of metabolic and hemodynamic responses and the corresponding pathological changes in neurovascular and neurometabolic coupling (Stiernman et al., 2023; Iadecola, 2017; Herman et al., 2025). Here [^18^F]FDG fPET offers a particular advantage as it is not dependent on neurovascular coupling or blood flow (Hahn et al., 2026), thereby enabling the disentanglement of distinct pathological alterations in patients with vascular risk factors. This also extends to the characterization of disease-related network alterations using simultaneous molecular and functional connectivity approaches. Here, the investigation of treatment-induced changes in neurotransmitter synthesis and release dynamics may offer particularly novel insights. The combination of neurotransmitter dynamics with functional connectivity further enables to assess the impact of neurotransmitter dysfunction in psychiatric disorders on brain network organization. fPET in general may also represent an attractive approach for neuromodulatory therapies such as deep brain stimulation as it contains fewer constraints in post-operative imaging than MRI. Here, reduction in methodological complexity will also facilitate broader application of fPET in clinical research. These included the validation of non-invasive assessment (Godbersen et al., 2024) and software availability (Hahn et al., 2025), thus only requiring a pump for the bolus+infusion radiotracer administration.

In sum, functional PET imaging is a rapidly growing field with promising opportunities to gain novel insights into brain energy metabolism and neurotransmitter signaling during stimulation as well as network organization at resting state, thereby providing complementary information to other techniques.

## Footnotes

1
https://neuroimaginglabsmuv.github.io/fPET-toolbox/
